# Income inequalities in multimorbidity prevalence in Ontario, Canada: a decomposition analysis of linked survey and health administrative data

**DOI:** 10.1186/s12939-018-0800-6

**Published:** 2018-06-26

**Authors:** Luke Mondor, Deborah Cohen, Anum Irfan Khan, Walter P. Wodchis

**Affiliations:** 1 Institute for Clinical Evaluative Sciences (ICES), G1 06 2075 Bayview Ave, Toronto, ON M4N 3M5 Canada; 2 Health System Performance Research Network (HSPRN), 155 College St 4th Floor, Toronto, ON M5T 3M6 Canada; 3 School of Epidemiology and Public Health, University of Ottawa, 600 Peter Morand Crescent, Ottawa, ON K1G Z53 Canada; 4 Institute of Health Policy, Management, and Evaluation (IHPME), University of Toronto, 155 College St 4th Floor, Toronto, ON M5T 3M6 Canada; 5 Institute for Better Health, Trillium Health Partners, 100 Queensway West, Mississauga, ON L5B 1B8 Canada

**Keywords:** Adult, Chronic disease, Comorbidity, Health status disparities, Ontario/ epidemiology, Prevalence, Socioeconomic factors, Trends, Advance equality in multimorbidity prevalence

## Abstract

**Background:**

The burden of multimorbidity is a growing clinical and health system problem that is known to be associated with socioeconomic status, yet our understanding of the underlying determinants of inequalities in multimorbidity and longitudinal trends in measured disparities remains limited.

**Methods:**

We included all adult respondents from four cycles of the Canadian Community Health Survey (CCHS) (between 2005 to 2011/12), linked at the individual-level to health administrative data in Ontario, Canada (pooled *n* = 113,627). Multimorbidity was defined at each survey response as having ≥2 (of 17) high impact chronic conditions, based on claims data. Using a decomposition method of the Erreygers-corrected concentration index (C_Erreygers_), we measured household income inequality and the contribution of the key determinants of multimorbidity (including socio-demographic, socio-economic, lifestyle and health system factors) to these disparities. Differences over time are described. We tested for statistically significant changes to measured inequality using the slope index (SII) and relative index of inequality (RII) with a 2-way interaction on pooled data.

**Results:**

Multimorbidity prevalence in 2011/12 was 33.5% and the C_Erreygers_ was − 0.085 (CI: -0.108 to − 0.062), indicating a greater prevalence among lower income groups. In decomposition analyses, income itself accounted more than two-thirds (69%) of this inequality. Age (21.7%), marital status (15.2%) and physical inactivity (10.9%) followed, and the contribution of these factors increased from baseline (2005 CCHS survey) with the exception of age. Other lifestyle factors, including heavy smoking and obesity, had minimal contribution to measured inequality (1.8 and 0.4% respectively). Tests for trends (SII/RII) across pooled survey data were not statistically significant (*p* = 0.443 and 0.405, respectively), indicating no change in inequalities in multimorbidity prevalence over the study period.

**Conclusions:**

A pro-rich income gap in multimorbidity has persisted in Ontario from 2005 to 2011/12. These empirical findings suggest that to advance equality in multimorbidity prevalence, policymakers should target chronic disease prevention and control strategies focused on older adults, non-married persons and those that are physically inactive, in addition to addressing income disparities directly.

**Electronic supplementary material:**

The online version of this article (10.1186/s12939-018-0800-6) contains supplementary material, which is available to authorized users.

## Background

An increasing number of adults in high-income settings have been diagnosed with multiple coexisting chronic conditions [1], also known as multimorbidity. This increased burden has created significant challenges in the effective provision of clinical care and has added pressures on health systems that traditionally provide highly specialized care for a single condition. Extensive research has shown that persons with multimorbidity have a greater risk of functional decline, cognitive decline and early mortality [2–4], are more frequent users of healthcare services, experience greater care fragmentation and longer hospital stays, and also incur higher healthcare costs [5–12].

Multiple studies from high-income settings have shown that multimorbidity is concentrated among persons from lower socio-economic ranks [13–17]. Multimorbidity may develop 10–15 years earlier among young and middle-aged persons in the most (vs. least) deprived areas [18]. While quantifying multimorbidity inequalities may help inform where policy action is needed, less is known about the relative contributions of determinants to socioeconomic disparities in multimorbidity [19]. These data are particularly useful to policymakers for informing future resource allocation and designing targeted interventions. Moreover, few studies have quantified longitudinal trends in measured inequalities in multimorbidity prevalence by income, education, or occupation, despite calls for action by federal agencies and professional organizations in many jurisdictions to reduce health disparities [20, 21].

To address these gaps, we undertook a comprehensive examination of inequalities in multimorbidity prevalence in Ontario, Canada. Our key research objectives were to: 1) quantify household income inequalities in multimorbidity prevalence among adults in Ontario, 2) identify the relative contribution of key determinants of multimorbidity to this inequality including socio-demographic, socio-economic, lifestyle and health system factors; and 3) assess whether these disparities were widening, and whether the drivers contributing to this inequality were changing over time.

## Methods

We used (cross-sectional) survey data and linked health administrative information that is routinely collected in Ontario. The use of this data was authorized under section 45 of Ontario’s Personal Health Information Protection Act, which does not require review by a Research Ethics Board. The study is reported according to the RECORD guidelines [22].

### Data and setting

Residents of Ontario, Canada aged 18 years and older that participated in any of four cycles of the Canadian Community Health Survey (CCHS) – 2005, 2007/08, 2009/10 and 2011/12 – and whose responses were linked to population-based health administrative databases were included in the study. Each survey was analyzed separately, unless otherwise stated (pooled cross-sections for longitudinal analysis).

The CCHS is administered by Statistics Canada to Canadians aged ≥12 years living in private dwellings, and is representative of 98% of the Canadian population. The CCHS does not include into its sampling frame persons living on Reserves or Crown Lands, institutionalized residents or members of the Canadian Forces. Detailed methodology of the survey and sampling strategy are described elsewhere [23, 24]. Permanent residents of Ontario (2011 population 12.8 million) are covered by a universal health insurance program that covers the costs for most physician and hospital services. Immigrants receive services after a three-month waiting period. Patient encounters with the healthcare system are recorded in administrative databases. Survey and administrative data were linked deterministically at the individual level using unique encoded identifiers and analyzed at the Institute for Clinical Evaluative Sciences (ICES) in Toronto, Ontario. The databases utilized in this study included the Ontario Health Insurance Program claims database (OHIP), Discharge Abstract Database (DAD), Ontario Drug Benefits database (ODB), Registered Persons Database (RPDB), and the Client Agency Program Enrolment database (CAPE) (see: https://datadictionary.ices.on.ca/Applications/DataDictionary/Default.aspx).

### Variables

We used health administrative data (OHIP, DAD, and ODB data) to determine the prevalence of 17 high-impact chronic conditions for each individual at time of their survey response. Consistent with previous studies of multimorbidity in Ontario [1, 12, 14, 25–27], these conditions were selected based on their economic impact and population burden in the general population. The 17 conditions included: acute myocardial infarction, asthma, cancers, cardiac arrhythmia, chronic coronary syndrome, chronic obstructive pulmonary disorder, congestive heart failure, diabetes, hypertension, mood and anxiety disorders, other mental illnesses (schizophrenia, delusions and other psychoses, personality disorders and substance abuse), osteoarthritis, osteoporosis, renal failure, rheumatoid arthritis and stroke (excluding transient ischemic attack). Where available, validated algorithms were applied to the health administrative data to ascertain cases, including AMI, asthma, COPD, CHD, dementia, diabetes and hypertension [28–34]. As in most other studies [18, 19, 35] we defined multimorbidity from this data as the co-occurrence of 2 or more (of these 17) conditions within the same individual, prevalent at the time of survey response.

We used household income from CCHS responses to measure socio-economic status (all respondents are asked: *“what is your best estimate of the total income received by all household members, from all sources, before taxes and deductions, in the past 12 months?”*). We focused specifically on (household) income as a marker of socioeconomic status to measure inequality because the rich-poor income gap is widening in most countries [36, 37], the strong association relating income to population health is well documented [38], and these gradients are potentially amenable to policy. Because of the sensitive nature of income reporting (resulting in missing or misclassified data), we used the imputed income responses provided by Statistics Canada. This method has been described extensively elsewhere [39]. These data were aggregated to quintiles for analysis and reporting.

Independent variables were selected a priori based on previous literature reporting associations with multimorbidity prevalence from similar high-income jurisdictions [13, 18, 40, 41]. Socio-demographic variables included age group (18–34 years, 35–49, 50–64, 65–74 and 75-plus), sex (men and women), rurality (urban, suburban or rural residence - based on the Rurality Index of Ontario [42], from RPDB data), marital status (married vs. divorced, separated, widowed or single), and immigrant status (born in Canada vs. not born in Canada). In addition to income, socio-economic variables (from CCHS data) included the respondent’s education level (no post-secondary vs. some post-secondary or higher). Health-related lifestyle characteristics included level of physical activity (active, moderately active, inactive), smoking status (heavy smoker, light smoker, former smoker, non-smoker) and body-mass index (BMI, underweight, normal weight, overweight, obese, following the classifications defined by the World Health Organization [43]). Health system variables included health region of the respondent’s residence (i.e., Local Health Integration Network [LHIN]) and enrolment in a primary model of care, which was used a proxy for access to primary care services (Family Health Group [FHG], Family Health Network [FHN], Family Health Organization [FHO], other, and not enrolled [CAPE data]) [44, 45]. Values of survey variables were selected based on the distribution of responses and to reflect meaningful categories. We excluded individuals from analysis if they were missing information (non-response) for any of these variables because there are currently no methods for combining the multiple imputation estimates specific to decomposition analyses.

### Statistical analyses

For each cycle, we transformed income data into cumulative rank probabilities (ridit scores) ranging from 0 (highest income) to 1 (lowest income), with values reflecting the midpoint of the cumulative proportion of the (weighted) population in each income group. Using this variable we quantified inequalities in multimorbidity for each CCHS cycle, first, using the Concentration Index (C) [46]. This relative measure of inequality considers the entire distribution of income, and can be written as:

$$ C=\frac{2}{n\mu}\sum \limits_{i=1}^n{y}_i{R}_i-1 $$

where C is the concentration index, *μ* is the (weighted) population mean of the outcome (here, multimorbidity prevalence), *y* is the outcome mean of the *i*th individual, and *R*_*i*_ is the individuals rank in the income distribution. Values range from −1 to + 1, corresponding to a concentration of the outcome (multimorbidity prevalence) among the poorer or wealthier population, respectively, where larger values (approaching −1 or + 1) indicate greater inequality, and a value of zero indicates equality. Because multimorbidity is dichotomous, we applied the Erreygers correction (C_Erreygers_), which multiplies C by 4 times the weighted mean of the health outcome [47]. Standard errors and 95% confidence intervals (CI) for C_Erreygers_ were derived using the methods described by O’Donnell et al. [48].

Wagstaff et al. [49] showed that C of a continuous health outcome can be decomposed into a set of determinants, a methodology that has since been extended to dichotomous variables [48, 50, 51]. In comparison to traditional analyses, this decomposition method allows for the explanation of the measured health inequality across the entire distribution of socio-economic status. To perform the decomposition, we specified a probit model with marginal effects [48], including all independent variables previously listed in the regression. Income itself is included as a determinant in the decomposition regression to prevent overestimation of the contribution of all other factors to measured inequality [52]. For any determinant to contribute to measured inequality, that determinant must be associated with the health outcome (i.e., have a non-zero regression coefficient) and also be unequally distributed by socio-economic status (i.e., have a non-zero concentration index). A positive (negative) contribution means that that determinant is associated with greater (lower) income inequality in multimorbidity. In addition to the proportional contribution of each determinant is a residual, representing the amount inequality in multimorbidity that cannot be explained by variation of each determinant across the income distribution. Negative residual values are possible, suggesting that determinants entered into the decomposition explain all measured inequality. We present decomposition results from the most recent survey, and describe differences in relative contribution of determinants to the baseline cycle.

Multiple sensitivity analyses were performed to assess the robustness of these decomposition analyses. Analyses were repeated using different reference categories in the multivariable probit model [50], performing the decomposition using a logit model approach (i.e., decomposing the natural logarithm of the odds of multimorbidity) [51], decomposing the Wagstaff-corrected C [53], and using a continuous log of household income term in the model (rather than quintiles). For each sensitivity analysis, we assessed any changes in the rank-order of the contribution of determinants measured inequality. Additionally, to ensure the measured inequality was robust to missing data, we re-calculated the C-Erreygers for each survey, including those with missing determinant data.

Lastly, to confirm changes in inequality over the study period, we quantified the relative index of inequality (RII) and slope index of inequality (SII). These measures are recommended for assessing trends in health disparities [54, 55]. First, we estimated the RII in multimorbidity prevalence within each cycle using a Poisson regression model [56] including covariates for ridit score, age group and sex. The exponential of the ridit score estimate is equivalent to the RII, which can be interpreted as the proportion of multimorbidity prevalence that differs between the highest and lowest incomes. An RII > 1 is indicative of pro-rich inequality on the relative scale (with larger values suggesting a greater concentration of multimorbidity among the poor). To asses trends, we used the methods outlined by Ernstsen et al. [57] using pooled survey data (with survey-specific ridit scores) and included covariates for ridit score, age group, sex, CCHS cycle and the 2-way ridit*cycle interaction. A positive (negative) and statistically significant interaction term is indicative of increasing (decreasing) inequality, on the relative scale. These methods were repeated using a linear probability model to determine the SII, reflecting the absolute difference in multimorbidity prevalence between the highest and lowest incomes. An SII > 0 is indicative of pro-rich inequality on the absolute scale. We also assessed trends in absolute inequality trends using pooled data. As a sensitivity test, we confirmed the statistical significance of these analyses adjusting for all measured determinants. We applied bootstrap sampling weights using balanced repeated replication (*n* = 500) to analyses. All data management was conducted using SAS Enterprise Guide version 6.1 (SAS Institute, Inc.) and all analyses were performed using Stata/MP version 13.1 (StataCorp).

## Results

**Fig. 1:**
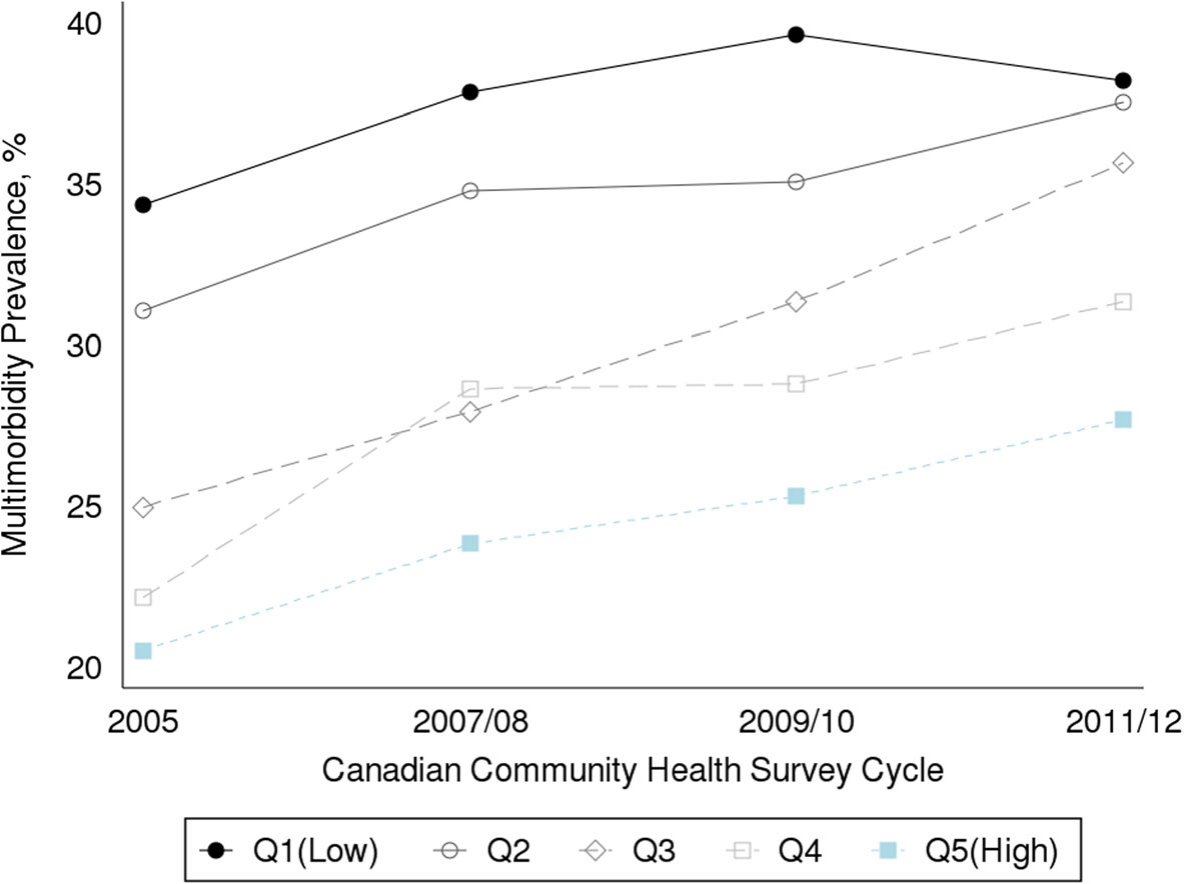
Longitudinal trends in multimorbidity by household income over four cycles of the CCHS

Of 134,395 Ontario respondents across 4 cycles of linked CCHS and administrative data, 122,002 (91% of sample) were adults, and 113,627 (93% of adults) had complete information across all variables for analysis. A higher proportion of adult respondents excluded (vs. included) from analyses were age 75+ years, female, from the lowest income quintile and had prevalent multimorbidity. Table 1 describes the characteristics of respondents included in analyses for each CCHS cycle. The numbers outside and inside the brackets correspond to sample counts and population weighted proportions, respectively. Crude multimorbidity prevalence increased steadily with each cycle, from 26.4% in the 2005 survey to 33.5% in the 2011/12 survey. Crude prevalence estimates by household income quintile are shown in Fig. 1, and by determinant in the Additional file 1: Table S2. Of note, prevalence in the lowest vs. highest household income quintiles, respectively, were 34.7% vs. 20.8% in 2005 and 37.8% vs. 27.6% in 2011/12.

**Table 1:** Characteristics of adult (age ≥ 18 years) CCHS survey respondents linked to health administrative data: 2005 to 2011/12

Variable	CCHS 2005	CCHS 2007–08	CCHS 2009–10	CCHS 2011/12
Total N:	28,412	29,632	28,388	27,195
Prevalent Multimorbidity:	9168 (26.4%)	11,156 (30.3%)	11,255 (31.7%)	11,390 (33.5%)
Age Group (years)				
18–34	7324 (29.6%)	6563 (29.0%)	6471 (28.8%)	5988 (28.8%)
35–49	7417 (32.4%)	7311 (30.3%)	6323 (29.2%)	5557 (27.2%)
50–64	7046 (22.2%)	8152 (24.4%)	7790 (25.3%)	7706 (26.4%)
65–74	3645 (9.1%)	4132 (9.4%)	4177 (9.3%)	4452 (10.1%)
75+	2980 (6.7%)	3474 (6.9%)	3627 (7.5%)	3492 (7.4%)
Sex				
Women	15,322 (51.2%)	15,960 (51.2%)	15,565 (51.3%)	14,965 (51.4%)
Men	13,090 (48.8%)	13,672 (48.8%)	12,823 (48.7%)	12,230 (48.6%)
Marital Status				
Other (Divorced, separated, widowed or single)	12,331 (35.4%)	12,537 (35.9%)	12,357 (36.6%)	12,150 (37.8%)
Married or common-law	16,081 (64.6%)	17,095 (64.1%)	16,031 (63.4%)	15,045 (62.2%)
Immigrant				
Born in Canada	22,691 (68.7%)	23,322 (67.0%)	22,460 (66.2%)	21,739 (67.6%)
Not born in Canada	5721 (31.3%)	6310 (33.0%)	5928 (33.8%)	5456 (32.4%)
Rurality				
Urban (RIO < 10)	14,877 (71.8%)	15,734 (72.2%)	15,057 (72.2%)	14,342 (72.6%)
Suburban (RIO 10–39)	8664 (20.3%)	9276 (19.7%)	8724 (20.3%)	8342 (19.7%)
Rural (RIO > 40)	4871 (7.9%)	4622 (8.0%)	4607 (7.5%)	4511 (7.7%)
Income (provincial quintile)				
Q1 (Low)	5617 (18.6%)	5190 (18.3%)	5079 (18.7%)	4460 (17.9%)
Q2	5719 (19.8%)	5793 (19.2%)	5431 (19.0%)	5140 (19.1%)
Q3	5305 (19.8%)	5700 (19.8%)	5749 (20.7%)	5825 (19.4%)
Q4	5776 (20.7%)	6385 (21.3%)	6005 (20.3%)	5648 (21.6%)
Q5 (High)	5995 (21.2%)	6564 (21.3%)	6124 (21.3%)	6122 (21.9%)
Education (Individual)				
No Post-Secondary Education	10,488 (32.4%)	10,612 (31.8%)	9893 (30.3%)	9651 (30.1%)
At least Some Post-Secondary Education	17,924 (67.6%)	19,020 (68.2%)	18,495 (69.7%)	17,544 (69.9%)
Physical Activity				
Active	7471 (26.0%)	7284 (23.7%)	7464 (26.4%)	7729 (28.1%)
Moderately Active	7249 (25.1%)	7500 (24.7%)	7189 (24.4%)	7099 (25.8%)
Inactive	13,692 (49.0%)	14,848 (51.6%)	13,735 (49.2%)	12,367 (46.1%)
Smoker				
Heavy smoker (At least 1 pack per day)	2233 (6.4%)	2079 (5.9%)	1809 (5.1%)	1619 (4.7%)
Light smoker (less than 1 pack per day)	4723 (16.4%)	4540 (15.9%)	4116 (14.8%)	3987 (15.5%)
Former smoker	7799 (24.2%)	8264 (23.0%)	7635 (21.7%)	7554 (22.6%)
Non-smoker	13,657 (53.1%)	14,749 (55.2%)	14,828 (58.4%)	14,035 (57.1%)
Body-Mass Index (BMI)				
Underweight (BMI < 18.5 kg/m2)	679 (2.8%)	652 (2.8%)	603 (2.6%)	625 (2.6%)
Normal weight (BMI 18.5–24.9 kg/m2)	12,558 (47.4%)	12,405 (45.3%)	11,888 (45.4%)	11,250 (44.7%)
Overweight (BMI 25–29.9 kg/m2)	9915 (34.0%)	10,530 (34.8%)	9955 (34.0%)	9486 (34.2%)
Obese (BMI > 30 kg/m2)	5260 (15.8%)	6045 (17.2%)	5942 (18.0%)	5834 (18.5%)
Local Health Integration Network (LHIN)				
Erie St. Clair	2102 (5.0%)	1985 (5.0%)	2030 (4.9%)	1933 (4.8%)
South West	3706 (7.4%)	3631 (7.3%)	3588 (7.0%)	3282 (7.4%)
Waterloo-Wellington	1709 (5.4%)	1715 (5.4%)	1729 (5.6%)	1570 (5.7%)
Hamilton Niagara Haldimand Brant (HNHB)	3642 (11.0%)	3404 (10.5%)	3380 (11.0%)	3284 (10.7%)
Central West	862 (5.9%)	1296 (7.3%)	1210 (5.8%)	1143 (5.8%)
Mississauga Halton	1378 (8.9%)	1437 (8.6%)	1369 (8.7%)	1410 (9.4%)
Toronto Central	1058 (9.7%)	1377 (9.0%)	1177 (9.2%)	1127 (9.7%)
Central	1632 (11.8%)	1779 (12.6%)	1657 (13.0%)	1487 (13.0%)
Central East	2759 (12.2%)	2904 (11.7%)	2537 (12.0%)	2471 (11.3%)
South East	1894 (3.9%)	1846 (3.8%)	1842 (3.8%)	1759 (3.7%)
Champlain	2853 (9.4%)	2814 (9.2%)	2753 (9.4%)	2784 (9.4%)
North Simcoe Muskoka	1059 (3.2%)	1448 (3.5%)	1310 (3.5%)	1287 (3.3%)
North East	2727 (4.5%)	2677 (4.5%)	2455 (4.3%)	2365 (4.2%)
North West	1031 (1.8%)	1319 (1.6%)	1351 (1.6%)	1293 (1.6%)
Primary Care Model Affiliation				
Family Health Group (FHG)	7068 (23.6%)	11,616 (42.7%)	7765 (35.0%)	5648 (27.7%)
Family Health Network (FHN)	2160 (5.3%)	4244 (9.2%)	1957 (4.0%)	1279 (2.4%)
Family Health Organization (FHO)	1026 (3.3%)	3414 (10.4%)	10,566 (30.6%)	13,292 (42.1%)
Not Enrolled	18,035 (67.3%)	8510 (33.1%)	6114 (24.9%)	5253 (23.2%)
Other Model	123 (0.4%)	1848 (4.6%)	1986 (5.5%)	1723 (4.6%)
Concentration Index^a^	− 0.114*	− 0.114*	− 0.111*	− 0.085*
(95% Confidence Interval)	(−0.132, − 0.096)	(− 0.133, − 0.094)	(−0.132, − 0.089)	(−0.108, − 0.062)

*p < 0.05

^a^Concentration Index, Erreygers-corrected

The measured C_Erreygers_ are shown in the last row of Table 1: values ranged from − 0.114 (CI: -0.132, − 0.096) in 2005 to − 0.085 (CI: -0.108, − 0.062) in 2011/12. The negative and statistically significant C_Erreygers_ indicates a greater concentration of multimorbidity among persons with low household income, although the smaller C_Erreygers_ value in the 2011/12 survey suggests that inequality was less pronounced.

Table 2 summarizes the results of the C_Erreygers_ decomposition of multimorbidity prevalence in the most recent (2011/12) and baseline (2005) surveys. Results from the 2007/08 and 2009/10 surveys are available in the Additional file 1: Table S3. Data include marginal effects from probit regressions, elasticities of each determinant (β_k_*x̅_k_/μ), the C_Erreygers_ of each determinant, and the relative contribution of each determinant to measured inequality. For example, for persons aged 75+ in the 2011/12 CCHS cycle: the difference in conditional probability of multimorbidity (vs. 18–34 age group) was 77.3%; this group represented 7.4% of the total population (from Table 1) but persons were concentrated in lower income levels (C_k_*(4*μ) = − 0.061); and their contribution to measured income inequality was 55.6%.

**Table 2:** Decomposition of the Erreygers-corrected Concentration Index of multimorbidity prevalence among adults in Ontario: 2005 vs. 2011/12

	CCHS 2005, *N* = 28,412				CCHS 2011–12, *N* = 27,195			
Variable	Marg. Effects	Elas.	C_k_	% Contr.	Marg. Effects	Elas.	C_k_	% Contr.
Age Group (years) [REF = 18-34y]								
35–49	0.15*	0.183	0.097	−12.7	0.161*	0.131	0.065	−12.2
50–64	0.339*	0.286	0.095	−28.2	0.366*	0.289	0.120	−51.6
65–74	0.489*	0.168	− 0.072	30.9	0.585*	0.177	− 0.044	29.9
v75+	0.637*	0.162	− 0.086	48.2	0.773*	0.17	− 0.061	55.6
Sex [REF=Women]								
Men	−0.066*	−0.121	0.123	7.1	−0.056*	−0.081	0.113	7.4
Marital Status [REF = Married]								
Other	0.039*	0.052	− 0.194	6.6	0.05*	0.057	− 0.258	15.2
Immigrant [REF=Born in Canada]								
Not born in Canada	−0.003	−0.003	− 0.225	−0.6	− 0.03*	−0.029	− 0.225	−7.8
Rurality [REF=Urban]								
Suburban	−0.019*	−0.015	0.042	0.7	−0.019	−0.011	0.066	1.5
Rural	−0.016	−0.005	0.018	0.3	−0.034*	−0.008	0.028	1.1
Income (quintile) [REF = Q5 (high)]								
Q1 (Low)	0.06*	0.042	− 0.605	31.9	0.083*	0.044	− 0.589	57.2
Q2	0.034*	0.025	− 0.341	10.1	0.047*	0.027	− 0.344	18.8
Q3	0.011	0.008	−0.027	0.3	0.06*	0.034	− 0.050	3.5
Q4	0.013	0.01	0.306	−3.4	0.03	0.019	0.299	−10.5
Education [REF=Some post-sec.]								
No Post-Secondary Education	0.001	0.001	−0.252	0.2	0.011	0.01	−0.242	3.2
Physical Activity [REF = Active]								
Moderately Active	0.028*	0.026	0.064	−1.6	0	0	0.067	0
Inactive	0.027*	0.051	− 0.159	3.8	0.048*	0.066	− 0.193	10.8
Smoker [REF=Non-smoker]								
Heavy smoker	0.039*	0.009	− 0.016	0.5	0.05*	0.007	− 0.030	1.8
Light smoker	0.021	0.013	−0.031	0.6	0.018	0.008	−0.068	1.4
Former smoker	0.038*	0.035	0.045	−1.5	0.055*	0.037	0.061	−4
BMI [REF=Normal weight]								
Underweight	−0.005	−0.001	− 0.021	−0.1	− 0.044	−0.003	− 0.024	−1.3
Overweight	0.049*	0.063	0.043	−1.8	0.072*	0.073	0.041	−3.4
Obese	0.154*	0.092	− 0.022	2.9	0.171*	0.095	− 0.002	0.4
LHIN [REF = Erie St. Clair]								
South West	−0.035*	−0.01	0.021	0.6	−0.061*	−0.013	0.008	0.6
Waterloo-Wellington	−0.102*	−0.021	0.022	2.0	−0.074*	−0.013	0.014	1.2
HNHB	−0.018	−0.007	− 0.008	−0.1	− 0.077*	−0.025	− 0.002	−0.2
Central West	−0.028	−0.006	− 0.033	−0.8	− 0.068*	−0.012	− 0.031	−2.4
Mississauga Halton	−0.04	− 0.014	0.008	0.3	−0.01	− 0.003	0.003	0
Toronto Central	−0.013	−0.005	− 0.018	−0.2	− 0.097*	−0.028	− 0.003	−0.3
Central	−0.042*	−0.019	− 0.021	−0.8	− 0.089*	−0.034	− 0.030	−3.1
Central East	−0.013	− 0.006	−0.018	− 0.2	−0.047	− 0.016	−0.027	−1.5
South East	−0.042*	−0.006	0.006	0.2	−0.051	−0.006	0.007	0.4
Champlain	−0.037*	−0.013	0.024	0.8	−0.042	−0.012	0.039	1.9
North Simcoe Muskoka	−0.051*	−0.006	0.011	0.5	−0.056	−0.006	0.016	1.1
North East	−0.048*	−0.008	− 0.008	−0.3	− 0.046	−0.006	0.008	0.4
North West	−0.075*	−0.005	0.001	0.1	−0.12*	−0.006	0.006	0.8
Primary Care Model [REF=None]								
Family Health Group	0.058*	0.052	0.029	−1.5	0.154*	0.128	− 0.042	7.5
Family Health Network	0.015	0.003	0.019	−0.3	0.104*	0.008	0.011	−1.3
Family Health Org.	−0.052*	−0.007	− 0.012	−0.6	0.105*	0.132	0.093	−11.5
Other Model	0.058	0.001	0.000	0.0	0.121*	0.017	0.015	−2.1
Sum (of C_Erreygers_)			−0.107	93.8			−0.093	108.6
Residual (unexplained)			−0.007				0.007	
Erreygers-corrected Concentration Index			−0.114				− 0.085	

*indicates statistically significant (*p* < 0.05) marginal effect derived from multivariable probit regression model

Elas. = Elasticity (β_k_ * x̅_k_ / μ); C_k_ = Erreygers-corrected Concentration Index of determinant k

Individual determinants with the largest contribution to the observed pro-rich inequality in the 2011/12 CCHS cycle included low household income (57.2 and 18.8% for quintiles 1 and 2, respectively), older age (55.6 and 29.9% for ages 75+ and 65–74 years, respectively), being divorced, separated, widowed or single (15.2%), and physical inactivity (10.8%). The relative contribution of each of these determinants to measured inequality increased from the decomposition analysis conducted using the 2005 CCHS survey data. For example, older ages (75+ years) were less concentrated among the low income (vs. 2005) but the elasticity (i.e., the % change in multimorbidity associated with a % change in the determinant) increased between cycles; for non-married persons, the elasticity increased, as did the concentration of non-married individuals among lower income groups. In contrast, variables that contributed negatively to inequality in the latter survey included being middle-aged (− 51.6% and − 12.2% for 50–64 and 35–49 years, respectively), having higher household income (− 10.5% for quintile 4), enrolment in a FHO (− 11.5%) and being born outside of Canada (− 7.8%). Of note, the growth in FHO enrolment between surveys (from 3.3 to 42.1%) corresponded with a greater concentration among higher household income (C_Erreygers_ from − 0.012 to 0.093); the opposite pattern was found for enrolment in a FHG (increased in enrolment from 23.6 to 27.7% and shift in C_Erreygers_ from 0.029 to − 0.042).

**Fig. 2:**
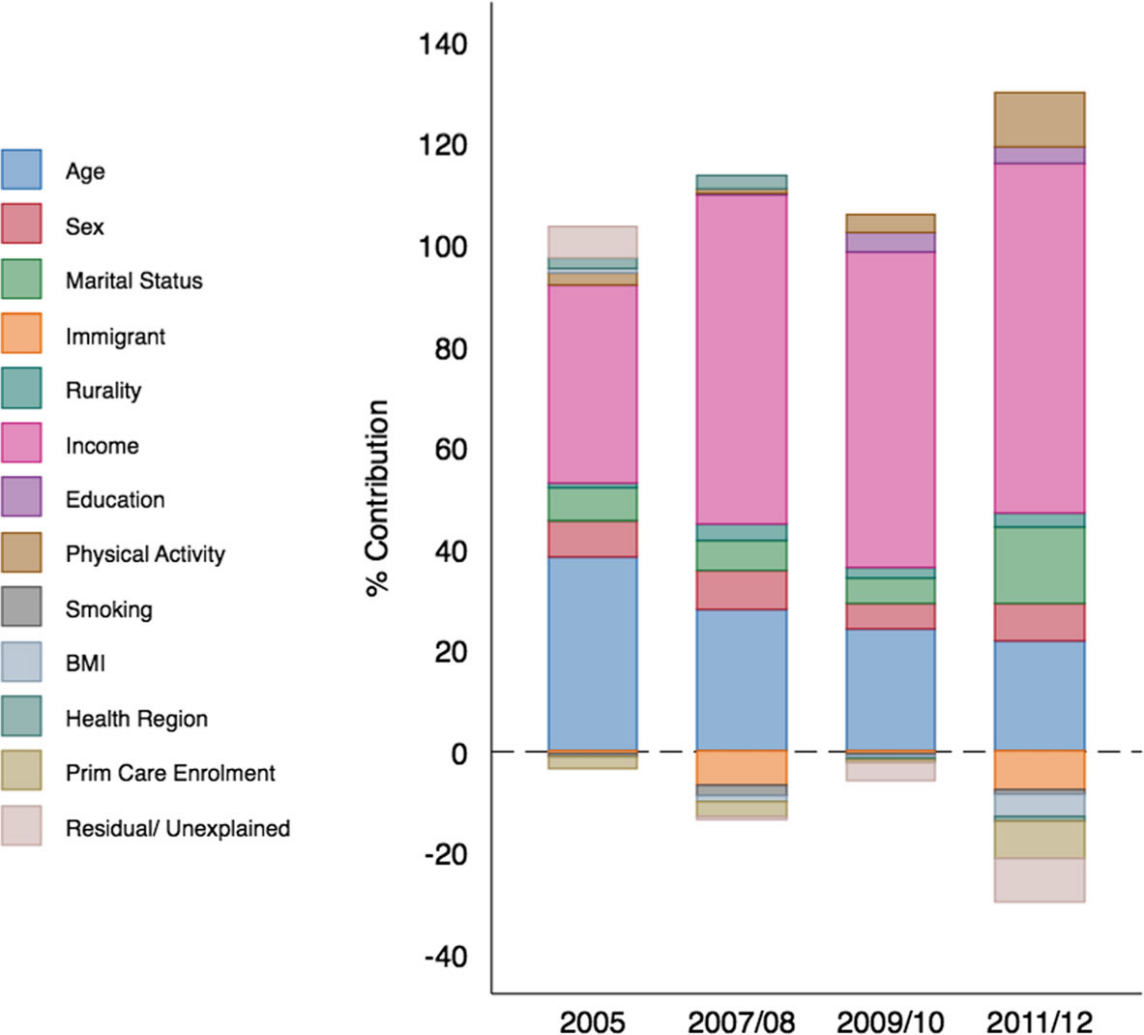
Summary of the relative contribution of key determinants to income inequality in multimorbidity in Ontario, 2005 to 2011/12

Aggregating these percentages by determinant (Fig. 2), income explained 69.0% of the measured inequality in 2011/12 (an increase of + 30.2% from 2005 CCHS survey), followed by age at 21% (− 16.5%), marital status at 15.2% (+ 8.6%), and physical activity at 10.9% (+ 8.6%). In contrast, determinants that contributed the largest to decreasing inequality were immigration at − 7.8 (− 7.2%) and primary care enrolment at − 7.4 (− 5.0%). In 2011/12, the modeled determinants explained − 0.093 of the total inequality (− 0.085), whereas in 2005 the determinants explained − 0.107 of the total inequality (− 0.114).

Table 3 shows the age-sex adjusted relative (RII) and absolute (SII) inequality measures for each survey, as well as the longitudinal trend analysis. The prevalence rate ratio (RII) in 2011/12 comparing high vs. low income quintile was 1.32 (CI: 1.19, 1.46) in 2011/12. The corresponding prevalence rate difference (SII) was 9.1% (CI: 5.7, 12.4%). In pooled regression analyses, longitudinal trends in inequality were not statistically significant over the study period on either the absolute or relative scale, suggesting no change in measured income inequality (p for interaction = 0.405 for RII and 0.443 for SII).

**Table 3:** Trends in income inequalities in multimorbidity prevalence among adults in Ontario, 2005 to 2011/12

CCHS Cycle:	2005	2007/08	2009/10	2011/12	Trend
Relative Index of Inequality					
RII (ridit score)	1.376* (1.233,1.535)	1.405* (1.280,1.543)	1.427* (1.290,1.579)	1.321* (1.191,1.466)	1.457* (1.288,1.649)
Age					
35–49	2.402* (2.093,2.756)	2.323* (2.007,2.689)	2.297* (2.011,2.624)	2.039* (1.742,2.387)	2.240* (2.081,2.412)
50–64	5.531* (4.865,6.287)	5.031* (4.408,5.741)	4.649* (4.151,5.207)	4.116* (3.585,4.726)	4.737* (4.437,5.057)
65–74	8.098* (7.164,9.155)	7.757* (6.825,8.816)	7.406* (6.620,8.287)	6.293* (5.553,7.131)	7.269* (6.843,7.721)
75+	10.287* (9.152,11.564)	9.017* (7.936,10.245)	8.761* (7.859,9.767)	7.667* (6.742,8.719)	8.762* (8.251,9.304)
Sex					
Male	0.832* (0.791,0.876)	0.853* (0.808,0.901)	0.911* (0.862,0.963)	0.884* (0.836,0.935)	0.873* (0.849,0.898)
CCHS Cycle					1.067* (1.036,1.099)
RII*Cycle					0.981 (0.937,1.027)
Constant	0.065 (0.052,0.074)*	0.076 (0.067,0.087)*	0.079 (0.070,0.089)*	0.098 (0.085,0.113)	0.067 (0.061,0.074)*
Slope Index of Inequality					
SII (ridit score)	0.082* (0.054,0.109)	0.099* (0.072,0.127)	0.110* (0.079,0.141)	0.091* (0.057,0.124)	0.082* (0.047,0.116)
Age					
35–49	0.100* (0.085,0.115)	0.114* (0.095,0.132)	0.120* (0.101,0.139)	0.113* (0.089,0.137)	0.112* (0.102,0.122)
50–64	0.316* (0.294,0.338)	0.338* (0.316,0.360)	0.330* (0.307,0.352)	0.332* (0.305,0.358)	0.329* (0.317,0.341)
65–74	0.509* (0.483,0.535)	0.581* (0.554,0.608)	0.592* (0.566,0.618)	0.571* (0.546,0.596)	0.564* (0.551,0.577)
75+	0.681* (0.657,0.706)	0.703* (0.677,0.729)	0.726* (0.703,0.749)	0.730* (0.703,0.756)	0.711* (0.699,0.723)
Sex					
Male	−0.048* (− 0.062,-0.035)	−0.048* (− 0.064,-0.032)	−0.030* (− 0.048,-0.013)	− 0.042* (− 0.060,-0.023)	−0.042* (− 0.050,-0.034)
CCHS Cycle					0.013* (0.005,0.021)
SII*Cycle					0.005 (−0.008,0.019)
Constant	0.053* (0.035,0.071)	0.056* (0.037,0.075)	0.048* (0.028,0.069)	0.079* (0.054,0.104)	0.026* (0.005,0.046)
Observations, N:	28,412	29,632	28,388	27,195	113,627

*p < 0.05

Data are regression coefficients from a Poisson regression (relative index of inequality, RII) or a linear probability model (slope index of inequality, SII)

Estimates and corresponding standard errors were estimated with bootstrap sampling weights using balanced repeated replication

For RII*Cycle, a statistically significant interaction with value > 1 (< 1) is indicative of increasing (decreasing) inequality on the relative scale over time; for the SII*Cycle, a positive (negative) and statistically significant interaction is indicative of increasing (decreasing) inequality on the absolute scale over time

These findings were robust to the defined sensitivity analyses (Additional file 1: Table S3).

## Discussion

This study confirms a moderate, statistically significant pro-rich household income gradient in multimorbidity prevalence in Ontario, Canada. This gap was observed on both relative and absolute scales, and has persisted from 2005 to 2011/12. Our results are consistent with measured wealth and socioeconomic inequalities in chronic conditions among adults observed in low and middle-income countries [19, 56, 58]. These studies considered only a small number of conditions in defining chronic disease burden. Our findings are also consistent with a study of multimorbidity (among 52 conditions) in deprived areas in the Basque Country of Spain [59]. Although socioeconomic differences in common chronic conditions among adult populations have been previously reported [60, 61], few studies have considered an aggregated count of multiple high impact chronic conditions or measured inequality with robust methods that account for the entire socio-economic distribution.

These findings highlight the unequal distribution of multimorbidity across socio-economic ranks. An important contribution of the current study to existing literature is the measurement of the relative contributions of multimorbidity’s determinants to measured income inequalities from a high-income setting. The decomposition method is useful for policy makers who seek to reduce health inequalities by targeting determinants that contribute the most to observed inequalities. Recently, Kunna et al. [19] decomposed wealth-related inequalities in multimorbidity prevalence (defined as 2 or more of 7 chronic conditions) among adults aged 50 years and older in China (middle-income setting) and in Ghana (low-income setting). Considering a similar set of measured determinants, the authors found that largest contributors to inequality were wealth, age, and education in China and BMI, wealth and rurality in Ghana. We found that the largest contributor to household income inequality in multimorbidity prevalence was income itself. This finding has been observed for similar decomposition studies of income-related inequalities in general health status from North America [62] and Europe [63]. Our data also show that the proportion of inequality in multimorbidity due to income increased over time. The growth in the rich-poor income gap in Canada, like may comparable OECD nations, has been well documented [37].

The strong negative association between increasing age and multimorbidity prevalence we observed is well established in existing literature [16]. The positive contribution of age to measured inequality in our study decreased steadily between cycles, mainly due to changes among the 50–64 year age group, who became more concentrated among higher household incomes. Among other socio-demographic variables, the contribution of marital status to measured inequality increased. Similar to age, where the change to the elasticity was minor, the increase in contribution was driven by a greater concentration of divorced, separated, widowed or single individuals in lower household income levels.

Physical activity was the strongest lifestyle factor contributing to observed inequalities in multimorbidity. We found that prevalence of multimorbidity increased over the study period (from 29.4 to 39.5%) among inactive persons in Ontario, and that inactivity was increasingly concentrated among lower incomes. Socioeconomic gradients in inactivity have been documented in other high-income jurisdictions [64], although less is known regarding changes across the socioeconomic distribution over time. Body-mass index had only a small negative contribution to inequality overall, and obesity in particular was relatively equally distributed across income. This contrasts findings by Hajizahen et al. [65], who report a small but significant concentration of obesity among the poor in Canada. Their data, however, exclude older adults (aged 65+ years). Our findings show that heavy and former smoking were unequally distributed across income but had small (although statistically significant) regression effects and small corresponding elasticities, also resulting in a minor contribution to inequality. The variability in the contribution of lifestyle determinants to inequalities in multimorbidity from previous work from low and middle-income jurisdictions [19] suggest that interventions meant to improve equality need to be targeted to local contexts. Other unhealthy lifestyle factors that are potentially amenable to public health intervention, such as healthy diet and alcohol consumption, were not included in our multivariable decomposition analyses since their relationships with multimorbidity prevalence are less certain [40].

The contribution of health system variables to overall inequality, including health region of residence and enrolment in a primary care model, were small. All participants of the study had universal access to medically necessary care; however, enrolment into a primary care model is voluntary. The negative contributions for this determinant in particular suggest that holding all else constant, persons enrolled in these models (particularly FHNs) are less likely to be multimorbid and of higher household income, which has been previously documented [44, 45]. Lastly, across all decomposition analyses, being born outside of Canada was negatively associated with multimorbidity prevalence (to varying effect sizes), and negatively contributed to multimorbidity inequality. A healthy immigrant effect for chronic disease incidence has been previously documented in Canada, although with diminishing associations as immigrants’ time in Canada lengthened [66, 67]. The effect of time since immigrating on socioeconomic inequalities in multimorbidity (and other health outcomes) is an avenue for future research.

The results from the decomposition were robust to multiple sensitivity analyses. Other strengths of the research include the use of a large sample size, survey weights that are reflective of Ontario’s population, and high survey response rates. Our results are likely to be generalizable to other high-income jurisdictions with comparable universal health care systems. Validated algorithms were also used to ascertain disease status; linked survey and administrative data enabled this inclusion. There are limitations of this research that are worth noting, however. Variables were defined using routinely-collected health administrative and survey data which are subject to known limitations [22, 23]. For health administrative data in particular, ascertainment of chronic disease is dependent having a place of residence and on health system contact (physician visits or hospital admissions), which are less likely among vulnerable, low-SES individuals. This would underreport multimorbidity prevalence and the overall measured inequality. Each survey is cross-sectional by design, and therefore, causality cannot be inferred from the data or by the decompositions. Inequality trends were assessed over a 7-year period from biennial cross-sectional surveys; the inclusion of additional surveys could be an avenue for future research, once linked to administrative data. Importantly, interpretation of decomposition results rely on a correct model specification; Pseudo R-squared values from the multivariable probit models were 0.22 (2005 CCHS) and 0.23 (2011/12 CCHS) suggesting the variables included in the regression explain only a portion of the variance observed in the outcome. Lastly, we were only able to include program enrolment as a marker of primary care access. However, a high-performing primary care sector can support equitable health outcomes by promoting seamless access to services across the care continuum, including preventive care and ongoing chronic disease management [68–70]. As such, other access-related determinants not measurable with our data may contribute to income inequalities in multimorbidity prevalence. Additional research that includes primary care capacities could help inform policymakers, and play a pivotal role for reducing overall disparities.

## Conclusions

This study confirms a pro-rich income gap in multimorbidity prevalence that has persisted over time. This gap is driven primarily by income, increasing age, marital status (not being married or common-law) and physical inactivity. Additional research to uncover the root causes of co-existing chronic conditions and to develop and evaluate the effectiveness of prevention strategies are warranted to Acknowledgements.

We thank IMS Brogan Inc. for use of their Drug Information Database.

## Additional file

Additional file 1: **Table S1.** Multimorbidity prevalence (standard deviation) by determinant. **Table S2.** Decomposition results from 2007/08 and 2009/10 CCHS surveys. **Table S3.** Sensitivity Results. (DOCX 49 kb)

## Abbreviations

BMI: Body mass index
C: Concentration index
CAPE: Client Agency Program Enrolment database
CCHS: Canadian Community Health Survey
C_Erreygers_: Erreygers-corrected concentration index
DAD: Discharge Abstract Database
ICES: Institute for Clinical Evaluative Sciences
LHIN: Local Health Integration Network
ODB: Ontario Drug Benefit database
OECD: Organisation for Economic Co-operation and Development
OHIP: Ontario Health Insurance Program claims database
RII: Relative index of inequality
SII: Slope index of inequality
