# Supplementary material for: Income inequalities in multimorbidity prevalence in Ontario, Canada: a decomposition analysis of linked survey and health administrative data
Source: Int J Equity Health. 2018 Jun 26;17:90. doi: 10.1186/s12939-018-0800-6 (PMC6019796; doi:10.1186/s12939-018-0800-6)
Supplement: Supplementary file 1 — Table S1. Multimorbidity prevalence (standard deviation) by determinant. Table S2. Decomposition results from 2007/08 and 2009/10 CCHS surveys. Table S3. Sensitivity Results. (DOCX 49 kb) [file 12939_2018_800_MOESM1_ESM.docx]

**Table S1. Multimorbidity prevalence (standard deviation) by determinant**

| **Variable** | | **CCHS  2005** | **CCHS  2007/08** | **CCHS  2009/10** | **CCHS  2011/12** |
| --- | --- | --- | --- | --- | --- |
| Age Group (years) | |  |  |  |  |
|  | 18-34 | 7 (0.4) | 8.5 (0.5) | 9.1 (0.5) | 10.7 (0.7) |
|  | 35-49 | 16.6 (0.7) | 19.4 (0.8) | 20.6 (0.8) | 21.5 (1) |
|  | 50-64 | 38.1 (1) | 41.6 (0.9) | 41.3 (1.1) | 43.1 (1.2) |
|  | 65-74 | 58.8 (1.3) | 67.2 (1.3) | 68.9 (1.2) | 68.2 (1.2) |
|  | 75+ | 76.7 (1.2) | 80.2 (1.1) | 82.9 (1.1) | 84.7 (1.1) |
| Sex | |  |  |  |  |
|  | Women | 29.6 (0.5) | 33.6 (0.6) | 34.2 (0.6) | 36.5 (0.7) |
|  | Men | 23 (0.5) | 26.8 (0.6) | 29 (0.6) | 30.3 (0.7) |
| Marital Status | |  |  |  |  |
|  | Other (Divorced, separated, widowed or single) | 25.2 (0.6) | 28.1 (0.6) | 29.5 (0.6) | 31.5 (0.7) |
|  | Married or common-law | 27.1 (0.5) | 31.5 (0.5) | 32.9 (0.6) | 34.7 (0.7) |
| Immigrant | |  |  |  |  |
|  | Born in Canada | 25.3 (0.4) | 29.6 (0.4) | 30.6 (0.5) | 33.1 (0.6) |
|  | Not born in Canada | 28.7 (0.9) | 31.6 (0.9) | 33.8 (0.9) | 34.2 (1) |
| Rurality | |  |  |  |  |
|  | Urban (RIO<10) | 25.8 (0.5) | 29.6 (0.5) | 31 (0.5) | 32.4 (0.6) |
|  | Suburban (RIO 10-39) | 27.3 (0.6) | 31.7 (0.7) | 33 (0.8) | 36.6 (0.9) |
|  | Rural (RIO>40) | 29.7 (0.8) | 32.9 (1.1) | 34.7 (1.1) | 36.1 (1.1) |
| Income (provincial quintile) | |  |  |  |  |
|  | Q1 (Low) | 34.7 (1.1) | 37.8 (1.2) | 39.2 (1.2) | 37.8 (1.2) |
|  | Q2 | 31 (1) | 34.7 (1) | 35 (1.2) | 37.3 (1.2) |
|  | Q3 | 24.4 (0.9) | 27.9 (0.9) | 31.2 (1.1) | 35.6 (1.1) |
|  | Q4 | 22.3 (0.7) | 28.5 (0.9) | 28.8 (0.9) | 30.6 (1.1) |
|  | Q5 (High) | 20.8 (0.8) | 23.9 (0.8) | 25.3 (0.9) | 27.6 (1) |
| Education (Individual) | |  |  |  |  |
|  | No Post-Secondary Education | 33.1 (0.7) | 37.7 (0.8) | 39.7 (0.9) | 40.3 (0.9) |
|  | At least Some Post-Sec Education | 23.2 (0.5) | 26.8 (0.5) | 28.2 (0.5) | 30.6 (0.6) |
| Physical Activity | |  |  |  |  |
|  | Active | 19.9 (0.6) | 25.7 (0.8) | 24.9 (0.8) | 25.7 (0.8) |
|  | Moderately Active | 27.2 (0.8) | 28.5 (0.8) | 29.7 (0.9) | 31.2 (1) |
|  | Inactive | 29.4 (0.6) | 33.2 (0.6) | 36.3 (0.7) | 39.5 (0.8) |
| Smoker | |  |  |  |  |
|  | Heavy smoker | 25.6 (1.4) | 30.4 (1.8) | 33.1 (1.9) | 37.5 (1.9) |
|  | Light smoker | 19.4 (0.8) | 21.1 (0.9) | 25.6 (1.1) | 26.3 (1.1) |
|  | Former smoker | 36.9 (0.8) | 43 (0.9) | 45.4 (0.9) | 47.3 (1) |
|  | Non-smoker | 23.9 (0.5) | 27.6 (0.6) | 28 (0.6) | 29.7 (0.7) |
| Body-Mass Index | |  |  |  |  |
|  | Underweight | 19.2 (2.1) | 23.9 (2.4) | 23.9 (2.8) | 19.7 (2.4) |
|  | Normal weight | 21 (0.5) | 23.3 (0.6) | 24.6 (0.7) | 26.2 (0.7) |
|  | Overweight | 28.3 (0.7) | 32.7 (0.7) | 33.7 (0.8) | 37.1 (0.9) |
|  | Obese | 39.7 (1.1) | 44.7 (1) | 46.8 (1.1) | 46.3 (1.2) |
| Local Health Integration Network (LHIN) | |  |  |  |  |
|  | Erie St. Clair | 30.5 (1.2) | 34.4 (1.1) | 34.3 (1.4) | 40.4 (1.9) |
|  | South West | 27.8 (1) | 29.3 (1) | 31.2 (1) | 33.2 (1.3) |
|  | Waterloo-Wellington | 19.5 (1.1) | 24 (1.3) | 23.5 (1.4) | 30.3 (2.2) |
|  | HNHB | 28.8 (0.9) | 31.4 (1) | 31.9 (1.1) | 36 (1.3) |
|  | Central West | 25.3 (2.2) | 30.5 (2) | 27.6 (1.7) | 30.7 (2) |
|  | Mississauga Halton | 24.7 (1.5) | 28 (1.5) | 30 (1.7) | 34.8 (1.9) |
|  | Toronto Central | 25.7 (1.8) | 24.5 (1.6) | 31.4 (1.7) | 26.8 (1.8) |
|  | Central | 24.4 (1.3) | 28.4 (1.5) | 32.3 (1.6) | 30.1 (1.9) |
|  | Central East | 27.6 (1.5) | 35 (1.4) | 31.8 (1.6) | 35.6 (1.7) |
|  | South East | 28.5 (1.3) | 38.3 (1.9) | 36.5 (1.7) | 36.8 (1.8) |
|  | Champlain | 26.2 (0.9) | 29.8 (1.3) | 33.7 (1.3) | 34.7 (1.4) |
|  | North Simcoe Muskoka | 27 (1.7) | 33.1 (1.7) | 34.7 (2) | 36.2 (1.8) |
|  | North East | 28.8 (0.9) | 33 (1.1) | 34.9 (1.3) | 38.1 (1.5) |
|  | North West | 25.8 (1.6) | 28.4 (1.5) | 33.8 (2) | 31.3 (1.9) |
| Primary Care Model Affiliation | |  |  |  |  |
|  | Family Health Group (FHG) | 34.9 (0.8) | 35.9 (0.7) | 36.1 (0.9) | 38.5 (1.1) |
|  | Family Health Network (FHN) | 29.2 (1.3) | 33 (1.1) | 31.8 (1.6) | 37.2 (2.1) |
|  | Family Health Organization (FHO) | 24 (1.6) | 30.4 (1.1) | 34.5 (0.7) | 36.6 (0.8) |
|  | Not Enrolled | 23.3 (0.5) | 20.9 (0.7) | 20.7 (0.8) | 20.9 (1) |
|  | Other Model | 33.1 (5.6) | 39.4 (2) | 37.2 (2.3) | 36.1 (2.3) |

**Table S2. Decomposition results from 2007/08 and 2009/10 CCHS surveys. Aggregated contributions are in bold**

|  |  | **CCHS 2007-08, N=29,632** | | | |  | **CCHS 2009-10, N=28,388** | | | |
| --- | --- | --- | --- | --- | --- | --- | --- | --- | --- | --- |
| **Variable** | | **Marg. Effects** | **Elas.** | **C_k_** | **% Contr.** |  | **Marg. Effects** | **Elas.** | **C_k_** | **% Contr.** |
| **Age Group** (years) [REF=18-34y] | |  |  |  | **28** |  |  |  |  | **23.9** |
|  | 35-49 | 0.162* | 0.162 | 0.07 | -10 |  | 0.166* | 0.153 | 0.052 | -7.8 |
|  | 50-64 | 0.372* | 0.3 | 0.111 | -36.3 |  | 0.359* | 0.287 | 0.12 | -39.1 |
|  | 65-74 | 0.574* | 0.177 | -0.058 | 29.3 |  | 0.588* | 0.172 | -0.053 | 27.9 |
|  | 75+ | 0.711* | 0.161 | -0.072 | 45 |  | 0.752* | 0.178 | -0.063 | 42.9 |
| **Sex** [REF=Women] | |  |  |  | **7.5** |  |  |  |  | **5.1** |
|  | Men | -0.066* | -0.107 | 0.128 | 7.5 |  | -0.049* | -0.076 | 0.116 | 5.1 |
| **Marital Status** [REF=Married] | |  |  |  | **6** |  |  |  |  | **5.2** |
|  | Other | 0.033* | 0.039 | -0.211 | 6 |  | 0.024* | 0.028 | -0.236 | 5.2 |
| **Immigrant** [REF=Born in Canada] | |  |  |  | **-6.8** |  |  |  |  | **-0.7** |
|  | Not born in Canada | -0.032* | -0.035 | -0.24 | -6.8 |  | -0.003 | -0.003 | -0.242 | -0.7 |
| **Rurality** [REF=Urban] | |  |  |  | **3.4** |  |  |  |  | **2.1** |
|  | Suburban | -0.034* | -0.022 | 0.085 | 2.6 |  | -0.028* | -0.018 | 0.079 | 2 |
|  | Rural | -0.035* | -0.009 | 0.026 | 0.8 |  | -0.005 | -0.001 | 0.028 | 0.1 |
| **Income** (quintile) [REF=Q5 (high)] | |  |  |  | **64.7** |  |  |  |  | **62.2** |
|  | Q1 (Low) | 0.113* | 0.069 | -0.598 | 59.6 |  | 0.102* | 0.06 | -0.608 | 55.8 |
|  | Q2 | 0.057* | 0.036 | -0.34 | 17 |  | 0.044* | 0.026 | -0.332 | 13.1 |
|  | Q3 | 0.022 | 0.014 | -0.04 | 0.8 |  | 0.038* | 0.025 | -0.032 | 1.1 |
|  | Q4 | 0.047* | 0.033 | 0.308 | -12.7 |  | 0.029* | 0.018 | 0.301 | -7.8 |
| **Education** [REF=Some post-sec.] | |  |  |  | **0.4** |  |  |  |  | **3.6** |
|  | No Post-Secondary Education | 0.002 | 0.002 | -0.257 | 0.4 |  | 0.017 | 0.016 | -0.236 | 3.6 |
| **Physical Activity** [REF=Active] | |  |  |  | **0.9** |  |  |  |  | **3.8** |
|  | Moderately Active | -0.013 | -0.011 | 0.065 | 0.7 |  | -0.005 | -0.004 | 0.081 | 0.4 |
|  | Inactive | 0.001 | 0.002 | -0.182 | 0.2 |  | 0.021 | 0.032 | -0.184 | 3.4 |
| **Smoker** [REF=Non-smoker] | |  |  |  | **-2** |  |  |  |  | **-0.2** |
|  | Heavy smoker | 0.044* | 0.009 | -0.022 | 0.9 |  | 0.048* | 0.008 | -0.025 | 1.1 |
|  | Light smoker | -0.002 | -0.001 | -0.051 | -0.1 |  | 0.029* | 0.013 | -0.064 | 1.7 |
|  | Former smoker | 0.055* | 0.042 | 0.059 | -2.8 |  | 0.054* | 0.037 | 0.062 | -3 |
| **BMI** [REF=Normal weight] | |  |  |  | **-1.1** |  |  |  |  | **-0.6** |
|  | Underweight | 0.039 | 0.004 | -0.024 | 0.8 |  | 0.015 | 0.001 | -0.022 | 0.3 |
|  | Overweight | 0.061* | 0.07 | 0.073 | -3.9 |  | 0.062* | 0.067 | 0.035 | -2 |
|  | Obese | 0.184* | 0.105 | -0.012 | 2 |  | 0.205* | 0.116 | -0.006 | 1.1 |
| **LHIN** [REF=Erie St. Clair] | |  |  |  | **2.7** |  |  |  |  | **-0.1** |
|  | South West | -0.053* | -0.013 | 0.021 | 1 |  | -0.034 | -0.008 | 0.019 | 0.6 |
|  | Waterloo-Wellington | -0.082* | -0.014 | 0.02 | 1.5 |  | -0.095* | -0.017 | 0.019 | 1.7 |
|  | HNHB | -0.048* | -0.017 | -0.005 | -0.2 |  | -0.05* | -0.017 | 0.005 | 0.2 |
|  | Central West | 0.003 | 0.001 | -0.043 | 0.1 |  | -0.043 | -0.008 | -0.036 | -1.4 |
|  | Mississauga Halton | -0.026 | -0.007 | 0 | 0 |  | -0.02 | -0.006 | -0.012 | -0.2 |
|  | Toronto Central | -0.072* | -0.022 | -0.009 | -0.6 |  | -0.004 | -0.001 | -0.001 | 0 |
|  | Central | -0.056* | -0.023 | -0.03 | -1.5 |  | -0.005 | -0.002 | -0.047 | -0.2 |
|  | Central East | 0.023 | 0.009 | -0.031 | 0.6 |  | -0.025 | -0.01 | -0.021 | -0.5 |
|  | South East | 0.045 | 0.006 | 0.003 | -0.1 |  | 0.01 | 0.001 | 0.01 | -0.1 |
|  | Champlain | -0.037 | -0.011 | 0.046 | 1.5 |  | 0.011 | 0.003 | 0.035 | -0.4 |
|  | North Simcoe Muskoka | -0.003 | 0 | 0.014 | 0 |  | 0.008 | 0.001 | 0.015 | -0.1 |
|  | North East | -0.035 | -0.005 | 0.002 | 0.1 |  | -0.015 | -0.002 | 0.011 | 0.2 |
|  | North West | -0.087* | -0.005 | 0.004 | 0.3 |  | -0.027 | -0.001 | 0.005 | 0.1 |
| **Primary Care Model** [REF=None] | |  |  |  | **-3.1** |  |  |  |  | **-0.6** |
|  | Family Health Group | 0.105* | 0.148 | 0.012 | -1.1 |  | 0.136* | 0.15 | -0.043 | 5.3 |
|  | Family Health Network | 0.06* | 0.018 | 0.041 | -2.2 |  | 0.048* | 0.006 | 0.021 | -0.9 |
|  | Family Health Org. | 0.026 | 0.009 | 0.023 | -0.5 |  | 0.085* | 0.082 | 0.082 | -6.3 |
|  | Other Model | 0.109* | 0.016 | -0.007 | 0.7 |  | 0.099* | 0.017 | -0.015 | 1.3 |
| Sum (of CE_rreygers_) | |  |  | -0.114 | **100.5** |  |  |  | -0.115 | **103.7** |
| Residual (unexplained) | |  |  | 0.001 | **-0.5** |  |  |  | 0.004 | **-3.7** |
| Erreygers-corrected Concentration Index: | | |  | -0.114 |  |  |  |  | -0.111 |  |
| * indicates statistically significant (p<0.05) marginal effect derived from multivariable probit regression model | | | | | | | | | | |
| Elas. = Elasticity (β_k_ * x̅_k_ / µ); Ck = Erreygers-corrected Concentration Index of determinant k | | | | | | | | | | |

**Table S3. Sensitivity Results**

**A) Erreygers-corrected concentration index values considering all respondents (including those excluded from the decomposition analysis due to missing information).**

| **Variable** | **CCHS 2005** | **CCHS 2007/08** | **CCHS 2009/10** | **CCHS 2011/12** |
| --- | --- | --- | --- | --- |
| C-Erreygers | -0.115* | -0.115* | -0.114* | -0.085* |
| 95% CI | (-0.133, -0.096) | (-0.134, -0.096) | (-0.134, -0.093) | (-0.107, -0.063) |
| Abbreviations: C-Erreygers=Erreygers corrected concentration index, 95% CI: 95% confidence interval | | | |  |

**B) Decomposition results, changing the reference category in the probit regression model. Values represent the relative contribution (and rank-order) of each determinant to measured inequality.**

| **Variable** | **CCHS 2005** | **CCHS 2007/08** | **CCHS 2009/10** | **CCHS 2011/12** |
| --- | --- | --- | --- | --- |
| Age Group | 38.2 (2) | 28 (2) | 23.9 (2) | 21.7 (2) |
| Sex | 7.1 (3) | 7.5 (3) | 5.1 (4) | 7.4 (5) |
| Marital Status | 6.6 (4) | 6 (4) | 5.2 (3) | 15.2 (3) |
| Immigrant | -0.6 (11) | -6.8 (12) | -0.7 (12) | -7.8 (12) |
| Rurality | 1 (8) | 3.4 (5) | 2.2 (7) | 2.6 (7) |
| Income Q (HH) | 38.8 (1) | 64.7 (1) | 62.2 (1) | 69 (1) |
| Education (Indiv) | 0.2 (9) | 0.4 (8) | 3.6 (6) | 3.2 (6) |
| Physical Activity | 2.3 (5) | 0.9 (7) | 3.8 (5) | 10.9 (4) |
| Smoker | -0.4 (10) | -2.1 (10) | -0.3 (9) | -0.7 (8) |
| Body-Mass Index | 1 (7) | -1.1 (9) | -0.5 (10) | -4.3 (10) |
| LHIN | 2 (6) | 2.8 (6) | -0.2 (8) | -1 (9) |
| Primary Care Model | -2.3 (12) | -3.1 (11) | -0.6 (11) | -7.5 (11) |
| Abbreviations: HH=household, Indiv=individual, LHIN=Local Health Integration Network | | | |  |

**C) Decomposition results, based on a logit regression model. Values represent the relative contribution (and rank-order) of each determinant to measured inequality.**

| **Variable** | **CCHS 2005** | **CCHS 2007/08** | **CCHS 2009/10** | **CCHS 2011/12** |
| --- | --- | --- | --- | --- |
| Age Group | 38.4 (2) | 25.8 (2) | 21.3 (2) | 17.8 (2) |
| Sex | 7.3 (3) | 7.5 (3) | 4.9 (4) | 6.8 (5) |
| Marital Status | 6.9 (4) | 5.8 (4) | 5 (3) | 13.6 (3) |
| Immigrant | 0 (10) | -6.3 (12) | 0.2 (8) | -6.5 (11) |
| Rurality | 1 (7) | 3.3 (5) | 2 (7) | 2.3 (7) |
| Income Q (HH) | 43.2 (1) | 65.5 (1) | 60.6 (1) | 65.8 (1) |
| Education (Indiv) | 0.3 (9) | 0.4 (8) | 3.5 (6) | 3.1 (6) |
| Physical Activity | 2.7 (5) | 1.1 (7) | 3.9 (5) | 10 (4) |
| Smoker | -0.5 (11) | -2 (10) | -0.3 (10) | -0.9 (9) |
| Body-Mass Index | 1 (8) | -1 (9) | -0.5 (11) | -4.1 (10) |
| LHIN | 2.1 (6) | 3 (6) | -0.1 (9) | -0.7 (8) |
| Primary Care Model | -2.5 (12) | -3.3 (11) | -0.6 (12) | -7.2 (12) |
| Abbreviations: HH=household, Indiv=individual, LHIN=Local Health Integration Network | | | |  |

**D) Decomposition results, using the Wagstaff-corrected concentration index. Values represent the relative contribution (and rank-order) of each determinant to measured inequality.**

| **Variable** | **CCHS 2005** | **CCHS 2007/08** | **CCHS 2009/10** | **CCHS 2011/12** |
| --- | --- | --- | --- | --- |
| Age Group | 38.2 (2) | 28 (2) | 23.9 (2) | 21.7 (2) |
| Sex | 7.1 (3) | 7.5 (3) | 5.1 (4) | 7.4 (5) |
| Marital Status | 6.6 (4) | 6 (4) | 5.2 (3) | 15.2 (3) |
| Immigrant | -0.6 (11) | -6.8 (12) | -0.7 (12) | -7.8 (12) |
| Rurality | 1 (8) | 3.4 (5) | 2.2 (7) | 2.6 (7) |
| Income Q (HH) | 38.8 (1) | 64.7 (1) | 62.2 (1) | 69 (1) |
| Education (Indiv) | 0.2 (9) | 0.4 (8) | 3.6 (6) | 3.2 (6) |
| Physical Activity | 2.3 (5) | 0.9 (7) | 3.8 (5) | 10.9 (4) |
| Smoker | -0.4 (10) | -2.1 (10) | -0.3 (9) | -0.7 (8) |
| Body-Mass Index | 1 (7) | -1.1 (9) | -0.5 (10) | -4.3 (10) |
| LHIN | 2 (6) | 2.8 (6) | -0.2 (8) | -1 (9) |
| Primary Care Model | -2.3 (12) | -3.1 (11) | -0.6 (11) | -7.5 (11) |
| Wagstaff-C | -0.146 | -0.135 | -0.128 | -0.096 |
| Abbreviations: HH=household, Indiv=individual, LHIN=Local Health Integration Network | | | |  |

**E) Decomposition results, using log of household income. Values represent the relative contribution (and rank-order) of each determinant to measured inequality.**

| **Variable** | **CCHS 2005** | **CCHS 2007/08** | **CCHS 2009/10** | **CCHS 2011/12** |
| --- | --- | --- | --- | --- |
| Age Group | 37.6 (1) | 27.5 (2) | 23.6 (2) | 21.8 (2) |
| Sex | 7.1 (3) | 7.5 (3) | 5 (3) | 7.6 (5) |
| Marital Status | 5.8 (4) | 4.4 (4) | 3 (6) | 14.2 (3) |
| Immigrant | -0.1 (10) | -5.8 (12) | -0.4 (10) | -6.8 (11) |
| Rurality | 1.2 (7) | 4 (5) | 2.8 (7) | 3.2 (7) |
| Log of HH Income | 34.1 (2) | 66.6 (1) | 66.9 (1) | 59.3 (1) |
| Education (Indiv) | 0.7 (9) | 0.3 (8) | 3.5 (5) | 3.7 (6) |
| Physical Activity | 2.4 (5) | 0.5 (7) | 3.7 (4) | 11 (4) |
| Smoker | -0.3 (11) | -1.8 (10) | -0.3 (9) | -0.8 (9) |
| Body-Mass Index | 1.1 (8) | -1.1 (9) | -0.4 (11) | -4.2 (10) |
| LHIN | 2.3 (6) | 3.5 (6) | 0.5 (8) | -0.4 (8) |
| Primary Care Model | -2.4 (12) | -3 (11) | -0.6 (12) | -7.5 (12) |
| Abbreviations: HH=household, Indiv=individual, LHIN=Local Health Integration Network | | | |  |

**F) Slope and relative index of inequality, adjusting for all measured determinants**

| **Variable** | **CCHS 2005** | **CCHS 2007/08** | **CCHS 2009/10** | **CCHS 2011/12** | **Trend** |
| --- | --- | --- | --- | --- | --- |
| Relative Index of Inequality |  |  |  |  |  |
| RII (ridit score) | 1.287* (1.146,1.446) | 1.383* (1.254,1.524) | 1.322* (1.186,1.474) | 1.257* (1.129,1.400) | 1.406* (1.241,1.594) |
| CCHS Cycle | -- | -- | -- | -- | 1.045* (1.013,1.077) |
| RII*Cycle | -- | -- | -- | -- | 0.973 (0.930,1.018) |
| Slope Index of Inequality |  |  |  |  |  |
| SII (ridit score) | 0.064* (0.034,0.093) | 0.093* (0.065,0.120) | 0.088* (0.056,0.121) | 0.075* (0.041,0.108) | 0.070* (0.035,0.104) |
| CCHS Cycle | -- | -- | -- | -- | 0.007 (-0.001,0.015) |
| SII*Cycle | -- | -- | -- | -- | 0.003 (-0.010,0.017) |
| Notes: inequality estimates adjusted for age, sex, marital status, immigration status, rurality, education, physical activity, smoking status, body-mass index, Local Health Integration Network, and primary care model affiliation (parameter estimates not shown for brevity) | | | | | |
| *denotes p<0.05 |  |  |  |  |  |
